# Applications of *Perilla frutescens* Extracts in Clinical Practice

**DOI:** 10.3390/antiox12030727

**Published:** 2023-03-16

**Authors:** Gigi Adam, Silvia Robu, Mihaela-Magdalena Flutur, Oana Cioanca, Ingrid-Andrada Vasilache, Ana-Maria Adam, Cornelia Mircea, Aurel Nechita, Valeriu Harabor, AnaMaria Harabor, Monica Hancianu

**Affiliations:** 1 Faculty of Pharmacy, “Grigore T. Popa” University of Medicine and Pharmacy, 16 Universitatii Street, 700115 Iasi, Romaniamihaela-magdalena_am_flutur@d.umfiasi.ro (M.-M.F.);; 2 Faculty of Medicine and Pharmacy, “Dunarea de Jos” University, 35 Al. I. Cuza Street, 800216 Galati, Romania; 3 Department of Obstetrics and Gynecology, ‘Grigore T. Popa’ University of Medicine and Pharmacy, 700115 Iasi, Romania

**Keywords:** *Perilla frutescens*, anti-inflammatory effect, anti-allergic effect, anti-oxidants, hypolipemiant

## Abstract

(1) Background: *Perilla frutescens* (L.) Britt. is an important pharmaceutical crop that remains a focus point for researchers worldwide due to its complex phytochemical constituents, medicinal effects, and nutraceutical properties. The literature data are based on animal and cell culture studies, so the clinical evidence for the therapeutic effects is poorly outlined. The aim of this review was to provide an updated and thorough understanding of *Perilla frutescens* applications in clinical practice using data derived from human studies, and to outline the potential directions and perspectives for further studies on this crop. (2) Methods: Medline, Embase, and Cochrane databases were used to find relevant studies. All interventional studies that evaluated the effect of *Perilla frutescens* in human subjects were assessed. (3) Results: The main perspectives that can be contoured from the presented literature evaluation are an important clinical effect of *Perilla frutescens* extracts on allergic rhinoconjuctivitis, especially in young populations, a potent hypolipemiant effect that, in conjunction with increased serum biological antioxidant potential, determines significant improvements in cognitive function and a wide variety of miscellaneous clinical effects that need further exploration. (4) Conclusions: Supplementary research is needed in order to demonstrate the therapeutic effects of *Perilla frutescens* in controlled clinical settings.

## 1. Introduction

*Perilla frutescens* (L.) Britt. (also referred to as Zisu in China), which belongs to the Lamiaceae family, has a widespread distribution globally, with a particularly high concentration in Asia, including China, Japan, Korea, and Vietnam [1]. In recent times, extensive research has been conducted regarding the phytochemistry and pharmacology of *Perilla frutescens* by several scientists. The plant’s isolated constituents include flavonoids, volatile oils, unsaturated fats, triterpenoids, phenols, and others [2,3].

*Perilla frutescens* is extensively utilized in traditional Chinese medicine (TCM) for the treatment of various illnesses, such as colds caused by wind cold, headaches, coughs, abdominal fullness and distension, and food poisoning from fish and crabs [1,4]. As a result of the many TCM practitioners’ recommendations for the leaves, stems, and seeds of this plant for a variety of medicinal objectives, the chemical contents and pharmacological properties of these sections of the plant have been both experimentally and scientifically verified. Furthermore, *Perilla frutescens* plays an essential role in various TCM-based prescriptions to augment the therapeutic effects of specific herbs in medical applications [5,6].

The compounds derived from *Perilla frutescens* exhibit various functions, such as anti-allergic, anti-inflammatory, antioxidant, anticancer, antibacterial, and antidepressant properties [1,7,8,9,10]. However, many of these properties were demonstrated in animal or in vitro studies. For example, Yang et al. investigated the antiallergic asthma effects of *Perilla* leaf extract (PLE) on ovalbumin (OVA)-sensitized mice, and showed that PLE treatment significantly attenuated airway inflammation by ameliorating lung pathological changes, inhibiting recruitment of inflammatory cells and the production of inflammatory cytokines in lung tissues and bronchoalveolar lavage fluid, as well as by reducing the level of immunoglobulin in serum [11]. On the other hand, several in vitro studies outlined the antitumoral effects of *Perilla frutescens* extracts on breast cancer [12], hepatocellular carcinoma [13], or lung adenocarcinoma [14].

In addition to these therapeutic effects, Kim et al. outlined the inhibitory effects of rosmarinic acid (RA), a compound of *Perilla frutescens* extracts, on adriamicin-induced apoptosis in H9C2 cardiomyocytes by inhibiting reactive oxygen species (ROS) generation and c-Jun N-terminal kinase (JNK), as well as extracellular signal-regulated kinase (ERK) activation [15]. These effects suggest a potential role of RA as a potential chemotherapeutic that inhibits cardiotoxicity in selected patients.

Several extracts have been shown to possess pharmacological actions beyond those already mentioned, including anti-human immunodeficiency virus (HIV)-1, neuroprotection, and anti-ischemic activity, as well as dermatological anti-aging or hepatoprotective effects, indicating further therapeutic utilizations [16,17,18,19,20].

The results from human studies regarding the effects of *Perilla frutescens* extracts are scarce, and data from these studies are heterogeneously reported. The aim of this review was to provide an updated and thorough understanding of *Perilla frutescens* applications in clinical practice using data derived from human studies, and to outline the potential directions and perspectives for further research on this plant.

## 2. Materials and Methods

We performed a literature search of published studies that evaluated the *Perilla frutescens* applications in clinical practice in MEDLINE, EMBASE, and Cochrane Library using synonyms of ‘*Perilla frutescens*’, ‘rosmarininc acid’, ‘apigenin’, ‘luteolin’, and Boolean operators AND/OR.

The time frame settled for this research was from inception up to the first of December 2022, with an English language restriction. Additional research consisted of manual screening of references cited in the evaluated papers in order to ensure that all relevant studies were included. Duplicates were removed using EndNote software version 20.4 (Clarivate, Philadelphia, PA, USA). The full-text papers were independently reviewed by two investigators (G.A. and A-M.A.) to establish their eligibility for the review. Any differences between the two were remedied by a third reviewer (S.R.) if a consensus could not be reached.

The inclusion criteria were represented by interventional studies, with a therapeutic study design that compared the therapeutic effects of at least one *Perilla frutescens* with a control group. We excluded opinion papers, studies that were published in another language than English, and case reports from the search.

Two investigators (G.A. and A-M.A.) retrieved data from the eligible studies separately using a standard process. Data concerning the first author, publication year, study design, characteristics of the population examined, number of cases and controls, and the therapeutic outcomes were obtained, and they were presented in a descriptive manner. Further, relevant in vitro and animal studies that evaluated the therapeutic effects were selected for a comprehensive perspective over the topic.

## 3. Evidence from In Vitro and Animal Studies Regarding the *Perilla frutescens* Effects

Many studies performed on cell cultures or on animal models outlined the anti-inflammatory, anti-allergic, antioxidant, and hypolipemiant effects of *Perilla frutescens*. Shin et al. found that aqueous extracts of *Perilla frutescens* inhibited mast cell-mediated immediate-type allergy responses in vivo and in vitro using rat models [21]. These extracts, when taken orally, exhibited inhibitory activity and anti-2,4, dinitrophenyl (DNP) induced immune local allergic responses that were dose-dependent.

Asada et al. discovered that a glycoprotein derived from an aqueous extract of *Perilla frutescens* considerably reduced mast cell inflammatory reaction and hyaluronidase and protein kinase C functions [22]. In a type I allergy rat model, following oral administration of rosmarinic acid (13 mg/kg) or matched dose of *Perilla frutescens* decoction (500 mg/kg) proffered a similar impact against mice ear-passive cutaneous anaphylaxis (PCA) reaction [23]. Another study, by Osakabe et al., investigated the anti-inflammatory properties of topical administration of rosmarinic acid on murine models [24]. Their results indicated a significant reduction in the neutrophil infiltration in skin samples after topical treatment, as well as an inhibition of the up-regulation of inflammatory enzymes and reactive oxygen species production.

In addition, a daily treatment with rosmarinic acid found in *Perilla frutescens* extract significantly reduced the percentage of eosinophils seen in bronchoalveolar lavage fluids as well as those found surrounding the animal’s airway in dermatophagoides farinae (Der f)-sensitized mice. Rosmarinic acid from these extracts had a considerable inhibitory effect on the production of interleukins 4 and 5, eotaxin in the lungs, and allergen-specific immunoglobulin G1 in the sensitized rats [25].

Rosmarinic acid is among the promising antioxidants derived from *Perilla frutescens* that has received the greatest research attention. It achieves this by inhibiting the generation of reactive nitrogen and oxygen species in lipopolysaccharide (LPS)-activated RAW264.7 macrophages [26], as well as the programmed cell death generated by adriamycin in H9c2 cardiomyocytes [15]. A summary of evidence from in vitro and animal studies regarding the *Perilla frutescens* effects is presented in Table 1.

## 4. Apigenin, Luteolin, and Rosmarinic Acid—Three Important Constituents of *Perilla frutescens*

**Figure 1:**
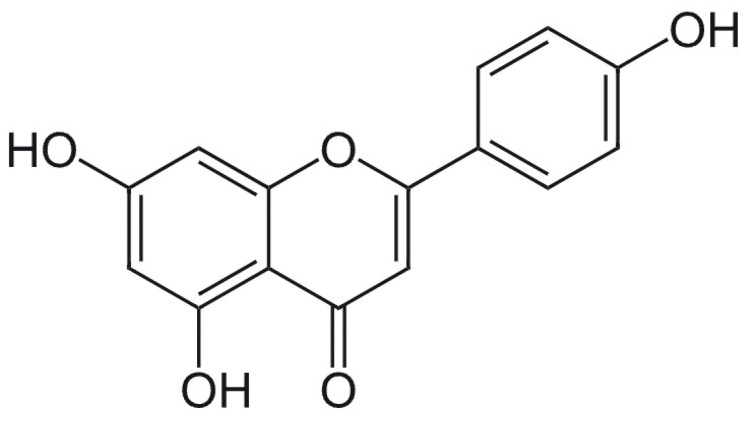
Chemical structure of apigenin.

Apigenin (4′,5,7-trihydroxyflavone) is a natural flavonoid commonly extracted from *Perilla frutescens* (Figure 1). The therapeutic properties of this compound are represented by antidepressant, anti-inflammatory, hepatoprotective, antithrombotic, antitumoral, antiaging, antioxidant, hypolipemiant, and anti-angiogenic effects [39,40,41,42,43,44,45].

**Figure 2:**
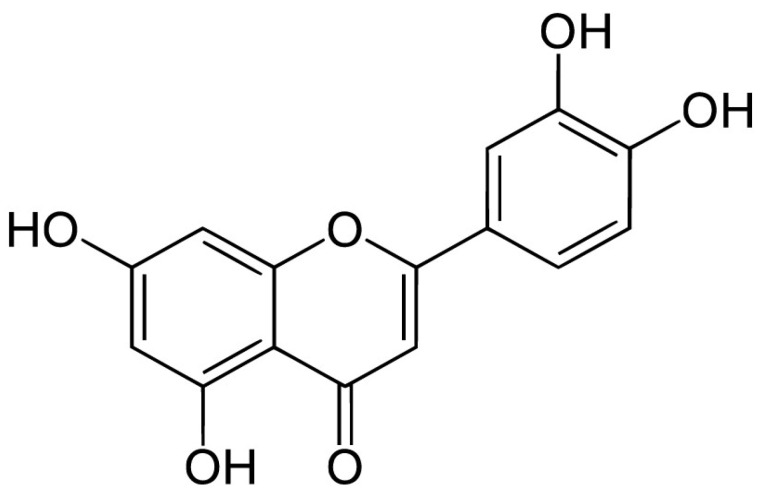
Chemical structure of luteolin.

Luteolin (3′,4′,5,7-tetrahydroxyflavone) (Figure 2) is another member of the flavones family, synthesized by the phenylpropanoid pathway in plants [46]. Its therapeutic effects are very similar to those of apigenin and include antioxidant, anti-inflammatory, hepatoprotective, antitumoral, and neuroprotective actions [47,48,49].

**Figure 3:**
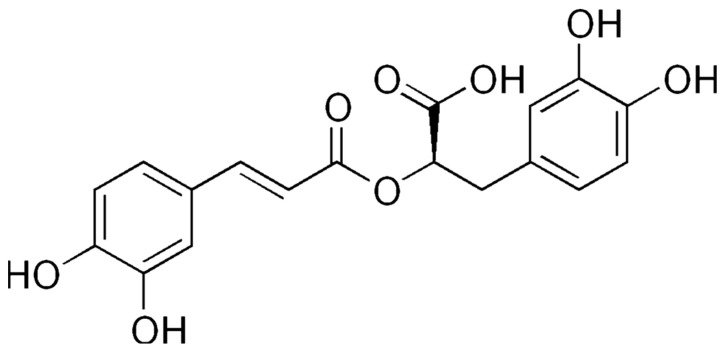
Chemical structure of rosmarinic acid.

Rosmarinic acid is an ester of caffeic acid and 3,4-dihydroxyphenyllactic acid, also known as (2R)-O-Caffeoyl-3-(3,4-dihydroxyphenyl)lactate (Figure 3) [50]. It is by far the most studied compound extracted from *Perilla frutescens* and has an extended spectrum of pharmacological effects demonstrated in animal and in vitro studies that ranges from anti-inflammatory and antioxidant to antitumoral and antimicrobial activities [2].

Apigenin and its glycosylated derivates were isolated from the seeds, stems, and leaves of *Perilla frutescens.* Zhou et al. evaluated phenolic compounds from *Perilla frutescens* var. *arguta* seed flour using column chromatography, and they determined a content of 9.88 μg/g apigenin [51]. Peng et al. used capillary electrophoresis with electrochemical detection for the identification of flavonoids in the leaves of *Perilla. frutescens* L. *Brit* and identified apigenin as one of the main compounds along with rosmarinic acid and luteolin [52]. Several other apigenin derivates represented by apigenin 7-O-glucuronide, apigenin 7-O-caffeoylglucoside, and apigenin 7-O-diglucuronide were isolated from the leaves of *Perilla frutescens* var. *frutescens* and var. crispa using spectophotometry [53,54].

Luteolin derivates, such as luteolin 7-O-diglucuronide, luteolin 7-O-glucoside, and luteolin 7-O-glucuronide, were isolated from the leaves of *Perilla frutescens* [53,54], while luteolin-5-O-glucoside was isolated from the seeds of this plant [51]. Rosmarinic acid, on the other hand, is one of the primary phenolic components in *Perilla frutescens* leaves and is particularly abundant from flowering to seeding [1,55].

Apart from rosmarinic acid, the seeds of *Perilla frutescens* comprise 30-dehydroxyl-rosmarinic acid-3-O-glucoside and rosmarinic acid-3-oglucoside [51]. A recent study by Deguchi et al. investigated the rosmarinic acid content from *Perilla frutescens* extracts and demonstrated a higher concentration of this compound in green varieties, in wild species, as well as in outdoor cultivated plants [56].

A literature review by Al-Khayri et al. provided a comprehensive perspective over the anti-inflammatory mechanisms of various flavonoids [57]. The authors outlined three main anti-inflammatory mechanisms for apigenin: anti TNF-α activity, cyclooxygenase-2 inhibition, as well as nitric oxide synthase inhibition.

Concerning their antioxidant capacity, it has been reported that extracts from *Perilla frutescens* seeds and leaves exhibit concentration-dependent antioxidant activity, which is attributed to phenolic compounds. Zhou et al. demonstrated that rosmarinic acid exhibited the highest 2,2-diphenyl-1-picryl-hydrazyl-hydrate (DPPH) radical-scavenging activity, followed by luteolin, luteolin-5-o-glucoside, and rosmarinic acid methyl ester. However, apigenin exhibited little DPPH-scavenging activity in this study [51].

Anti-inflammatory and antioxidant properties of luteolin were proven in animal studies that suggested inhibition of nitric oxide production [31], mast cell degranulation, and anti TNF-α activity as underling mechanisms for these effects [58]. Moreover, it was demonstrated that both apigenin and luteolin extracted from *Perilla frutescens* (L.) *Britt* act as potential monoamine transporter activators, in a similar way to antidepressant drugs [59].

Zhang et al. investigated how apigenin regulates cholesterol metabolism in rats and found that apigenin extracts enhanced hepatic messenger ribonucleic acid (mRNA) expression of 3-hydroxy-3-methylglutaryl-coenzyme A reductase (HMG-CoAR), cholesterol 7 alpha-hydroxylase (CYP7A1), and low-density lipoprotein receptor (LDL-R) [44].

The antiallergic and anti-inflammatory effects of rosmarinic acid are a result of reduced vascular permeability and leukocyte migration, moderation of cytokine and chemokines secretion, as well as an inhibition of specific antibody production [25,29]. Although the proposed mechanisms of action of rosmarinic acid are heterogeneously reported in the literature, several studies outlined, as potential effects, a reduction in the generation of reactive oxygen species, in the mRNA expression levels of various interleukins (IL-1β, IL-6, IL-8), as well as TNF-α and COX-2 inhibition [60,61,62].

## 5. Anti-Inflammatory and Antiallergic Effects

Inflammation is characterized by the overproduction of specific pro-inflammatory genes and cytokines. The anti-inflammatory properties of PLE have been outlined in animal studies through the down-regulation of mRNA expression, translation and transcription of pro-inflammatory mediators, as well as the inhibition of ERK 1/2, JNK, p38, and nuclear factor-κB (NF-κB) signaling [63].

It has been demonstrated that the total flavonoids found in *Perilla frutescens* have significant anti-inflammatory properties. These properties are represented by a reduction in vascular permeability, a suppression of the production of inflammatory cytokines, an enhancement in the scavenging of oxygen free radicals, and anti-lipid peroxidation attributes [64].

*Perilla frutescens* seeds contain fatty acids that have been shown to have anti-inflammatory properties. These fatty acids are able to exert this action by possibly reducing the formation of inflammatory lipid mediators, platelet activating factor (PAF), and leukotrienes (LTs) [23,65]. The fixed oil of *Perilla frutescens* has been demonstrated to be effective in treating reflux esophagitis due to its lipoxygenase inhibitory, histamine antagonist, anticholinergic, and antioxidant effects [66].

In addition, it has been shown that a variety of the chemicals extracted from the *Perilla frutescens* plant had anti-inflammatory activities. For instance, it has been demonstrated that the flavonoid compound luteolin has therapeutic effects in neuro-inflammatory illnesses by reducing the expression of inducible nitric oxide synthase (iNOS) [31]. Rosmarinic acid has also been demonstrated to have highly effective inhibitory activity on tumor necrosis factor alpha (TNF-α) and also in endothelial protein C receptor (EPCR) shedding by blocking TACE (TNF-α-converting enzyme) production [34]. Additionally, rosmarinic acid has the potential to be an effective therapeutic option for treating some inflammatory-related maladies by hindering the high-mobility group box 1 (HMGB1) signaling pathway [29].

A multicentric, randomized, double-blind, parallel-group, placebo-controlled study that evaluated the efficacy and safety of Lertal (a mixture of *Perilla* extract, quercetin, and Vitamin D3) as an add-on treatment in 146 children with allergic rhinoconjunctivitis demonstrated that this treatment was able to significantly prevent the occurrence of clinical worsening, and it was safe in AR (allergic rhinitis) poly-allergic children [67]. Intriguingly, this beneficial effect was visible in the second phase of the active treatment, during the third and fourth week, when certain patients, following an initial response to pharmacological treatment, displayed a worsening of symptoms. Moreover, a recent study by Shen et al., that employed the association rule Apriori algorithm for the data analysis of the medication regime and the therapeutic effect of traditional Chinese remedies for the treatment of allergic rhinitis in children, demonstrated that, when compared with the conditions before treatment, the children’s symptoms with allergic rhinitis were significantly diminished after a full regimen with traditional Chinese medicine compounds (*p* = 0.05), specifically *Perilla frutescens* extracts [68].

Marseglia et al. conducted a Phase II multicentric randomized, parallel-group controlled study to assess the effectiveness and acceptability of Lertal in countering allergy rhinitis (AR) flare ups in children after the curative treatment phase was completed [69]. The results demonstrated that such treatment was able to significantly reduce the risk of AR exacerbation, the duration, and the use of rescue medications after the suspension of the one-month antihistamine treatment. The observation period without AR symptoms among Lertal-treated children was significantly greater and constituted an important finding. This result has clinical significance of the utmost importance because it highlights the potential of reducing the need of drugs, particularly after a lengthy antihistamine treatment (i.e., four weeks). AR cannot be healed by drugs, even if they are taken for extended periods of time. Consequently, limiting the usage of pharmaceutical treatment is especially important in children. In addition, this result merits examination from a pharmacoeconomic standpoint. A longer timeframe without the presence of specific symptoms would determine a significant reduction in drug consumption. Additionally, a longer timeframe without symptoms would be strongly correlated with a better quality of life, since it is widely recognized that the intensity of allergic rhinitis affects this aspect.

For the adult population, the efficacy of Lertal was supported by studies with much smaller cohorts of patients. For example, an open clinical study of 23 subjects with at least one year history of allergic rhinitis and positive skin prick test or RAST to *Parietaria officinalis* pollen demonstrated a significant reduction in overall symptoms (sneezing, rhinorrhea, nasal obstruction, ocular itching, lacrimation, and congestion of the conjunctiva), symptom scores, and in the use of anti-allergic drugs. No noteworthy side effects were recorded in this study, and all patients finished it with good compliance [70].

A randomized, double-blind, placebo-controlled trial, conducted by Osakabe et al. on 29 subjects, assessed the anti-inflammatory and anti-allergic effects of various doses of Rosmarinic acid (50 or 200 mg per day) for patients affected by AR, who received the treatment or placebo for a 21-day timeframe [24]. The authors reported a significant reduction in symptoms, such as itchy nose, watery or itchy eyes, as well as an important reduction in numbers of neutrophils and eosinophils identified in the nasal lavage fluid. The second phase of the trial consisted of an animal study that evaluated the effects of *Perilla frutescens* extract, rosmarinic acid, and luteolin on edema formation in TPA (12-O-tetradecanoylphorbol-13-acetate)-induced rats.

The anti-inflammatory properties of rosmarinic acid were evaluated in a clinical trial of 21 subjects with moderate atopic dermatitis of the elbow’s flexure [71]. Rosmarinic acid (0.3%) cream was applied on the affected zone twice daily, and the symptoms and severity of the disorder were objectively evaluated using clinical examination and Severity Scoring of Atopic Dermatitis (SCORAD) index after 8 weeks of treatment. The authors reported a significant improvement in erythema and a reduction in transepidermal water loss on antecubital fossa, as well as a significant improvement in self-reported symptoms, such as dryness and pruritus.

The proposed molecular mechanism of this effect was an inhibition in TNF-α-induced production of C-C motif chemokine 11 (CCL11) and C-C chemokine receptor type 3 (CCR3), mediated through an inhibition in I-kappa-B-kinase (IKK-β) [71,72]. A summary of the anti-inflammatory and antiallergic properties of *Perilla frutescens,* as derived from studies on human subjects, is presented in Table 2.

## 6. Antioxidant and Hypolipemiant Effects

Consumption of so-called functional foods and nutraceuticals has been linked to a lower risk of cancer, cardiovascular disease, and metabolic disorders in epidemiological, clinical, and nutritional studies. According to the 2,2-diphenyl-1-picryl-hydrazyl-hydrate (DPPH) radical assay and the 2,20-azino-bis (3-ethylbenzothiazoline-6 sulphonic acid) (ABTS) radical cation assay, extracts from the seeds and leaves of *Perilla frutescens* exhibit concentration-dependent antioxidant activity [51].

From another perspective, apigenin and luteolin were the major elements in the total flavonoid extract of *Perilla frutescens* (TFP), which were able to reduce dyslipidemia, lipid deposition in adipose tissues, and serum concentrations of triacylglycerols, total cholesterol, and LDL cholesterol, while simultaneously increasing HDL cholesterol in rats that were fed a high-fat diet [73]. By preventing lipid peroxidation through lowering blood malondialdehyde (MDA) levels, TFP was able to minimize oxidative stress in hyperlipidemic rats and to increase the levels of antioxidant enzymes, such as superoxide dismutase (SOD) and glutathione peroxidase (GPx).

Oxidized low-density lipoprotein (Ox-LDL) has been associated with an increased risk of developing atherosclerosis. As a result, decreasing Ox-LDL levels in the blood via nutritional approaches is an important method for avoiding cardiovascular complications in targeted patients. A randomized placebo-controlled human interventional study evaluated the effects of *Perilla frutescens* leaf powder (PLP) over the Ox-LDL serum levels, biological antioxidant capacity, and blood pressure measured at home [74]. When compared with the control group, the PLP group had significantly reduced serum Ox-LDL concentration, significantly higher alteration in the biological antioxidant potential, and significantly lower levels of alpha-linolenic acid in the erythrocyte plasma membrane. Additionally, the PLP group had a substantial decrease in the systolic blood pressure.

A prospective study by Saita et al. investigated the effects of red and green *Perilla frutescens* extracts on LDL oxidation and antioxidant enzyme expression in vivo and in human subjects [38]. The scientists showed that both green and red varieties were rich in polyphenol compounds and had significant 1,1-diphenyl-2-picrylhydrazyl radical scavenging properties. In addition, both types of *Perilla frutescens* contained a large number of free radicals. In vitro testing showed that *Perilla frutescens* had a significant inhibitory effect on azo-radical-induced LDL oxidation as well as endothelial-cell-mediated LDL oxidation. In addition, the levels of mRNA and protein synthesis of antioxidant enzymes were dramatically raised in endothelial cells after treatment with the red variety of *Perilla frutescens*. After oral administration of red *Perilla frutescens*, the LDL oxidation lag times of the participants were significantly lengthened, whereas the production of lipid peroxide and the electrophoretic mobility of LDL were significantly reduced. According to these findings, *Perilla frutescens*, and particularly the red form of this plant, has a significant level of antioxidant activity, and it successfully inhibited the oxidation of LDL.

Plasma lipoproteins have been proposed as possible polyphenol transporters. After consuming *Perilla frutescens* extracts, polyphenols may be taken into the circulation and integrated into LDL, according to in vitro studies [75,76]. In addition, it must be considered that *Perilla frutescens* contains a variety of antioxidants, including polyphenols, vitamin E, and carotenoids [77,78,79]. Certain hydrophilic antioxidants link to phospholipids or proteins on the LDL periphery, while hydrophobic antioxidants attach nearer to the LDL nucleus, according to previous research [80,81]. On the basis of these previous studies, we may hypothesize that the combined effect of hydrophilic and hydrophobic antioxidants in *Perilla frutescens* extracts may have reduced lipid oxidation.

Age-related losses in memory and reasoning are linked to decreased plasma antioxidative capacity and increased oxidative stress, suggesting that the brain is particularly vulnerable to shifts in oxidative equilibrium [82,83]. There is a strong correlation between oxidative damage and cognitive decline, and current findings suggest that boosting antioxidant capacities may prevent cognitive decline in the elderly [84,85,86].

*Perilla frutescens* seeds oil (PO) administration alone or in conjunction with *Anredera cordifolia* leaf (AC) powder was tested for its influence on cognitive performance in healthy Japanese seniors in a randomized, double-blind, parallel-armed intervention experiment [87]. Participants were randomly assigned to receive either 1.47 mL of PO per day or a combination of 1.47 mL of PO and 1.12 g of AC powder per day. Twelve months after starting treatment, those in the PO-AC group performed better on the cognitive index than those in the PO group. Plasma levels of triglycerides, glucose, and N-(epsilon)-carboxymethyl-lysine (CML), an advanced end-product of glycation and biochemical marker of oxidative stress levels, were reduced, while serum levels of ALA and eicosapentaenoic acid were increased, all of which were associated with the beneficial effects of combined supplementation on cognitive function. After 12 months of treatment, there was a significant negative association between serum CML levels and the effects of combination supplementation on cognitive performance.

Hashimoto et al. conducted another randomized, double-blind, parallel-arm trial on the effects of PO supplements on cognitive performance in 49 healthy Japanese seniors, taking either PO alone or PO plus nobiletin-rich air-dried immature ponkan powder. Cognitive index scores were considerably higher in patients who received a combination of supplements. Improved cognitive performance was associated with elevated amounts of ALA and docosahexaenoic acid in erythrocyte membranes, brain-derived neurotropic factor (BDNF) in the blood, and biological antioxidant capacity [88]. Their results indicated that the group who received the combination of supplements showed significantly higher cognitive index scores. Improved cognitive performance was associated with elevated amounts of ALA and docosahexaenoic acid in erythrocyte membrane (BDNF) concentration in the blood, and biological antioxidant capacity.

A similar study design was employed by Hashimoto et al., in a cohort of 75 elderly patients, between 64 and 84 years, that aimed to assess the impact of dietary *Perilla frutescens* seed oil intake on cognitive functions and mental health [89]. The authors measured cognitive function, the presence of depression and apathy using standardized scales, as well as fatty acid profile of the red blood cell plasma membranes (RBC-PMs) and serum biochemical parameters. Their results indicated that 12 months after the experiment ended, the treatment group had considerably greater blood biological antioxidant capacity and α-linolenic acid content in the RBC-PM than the control group did. Further, after 12 months, those in the treatment group showed a trend toward enhanced cognitive function and reduced apathy.

Similar results were found in a 12-month randomized, double-blind, placebo-controlled study on 75 adult patients, which compared the effects of *Perilla frutescens* oil versus placebo treatment on the mental condition [90]. Serum biochemical markers were measured initially and again after 12 months of therapy, and the Zung Self-Rating Depression Scale (SDS) as well as the Apathy Scale were used to evaluate mental health. The authors reported that the treatment group showed statistically significant improvements in both depression and apathy scores. At 12 months, the treatment group had lower levels of serum monoamines (norepinephrine and serotonin) than the control group. Moreover, they observed an increase in α-linolenic acid levels in in the RBC-PM, which paralleled the improved mental state found in subjects who received the *Perilla frutescens* oil. The authors hypothesized that the shifts in catecholamine concentrations facilitated a neuro-adaptive process that struck a dynamic equilibrium among both serotonergic and noradrenergic activity in the brain that would justify the improvements in depression and apathy scores for the treatment group.

A lower risk of developing severe depression, postpartum depression, and manic-depressive illness has been linked to higher intakes of ω-3 polyunsaturated fatty acids (PUFAs), according to recent research, although these conclusions are based on low-quality data [91,92,93]. Eicosapentaenoic acid (EPA) and docosahexaenoic acid (DHA), two types of ω-3 PUFAs, were associated with a reduction in depression symptoms and prevention of their onset [94,95,96]. Additionally, the theory that lowering linoleic acid (LA) consumption and increasing α-linolenic acid (ALA) intake would decrease depression risk was presented in a few studies [97,98].

The oil extract of *Perilla frutescens* seeds contains a large quantity of ALA. Lower amounts of ω-3 PUFAs or ALA instead of EPA or DHA in red blood cell (RBC) membranes may be strong predictors of cognitive impairment in older persons with recurrent depression [99]. Moreover, previous research has shown that ω-3 PUFA supplementation may lower corticosterone levels in rats under stress due to its anti-inflammatory characteristics [100]. In another animal study, McNamara et al. discovered that rats given an ALA-deficient diet had lower levels of tryptophan hydroxylase (TPH)-2 mRNA expression, which is an enzyme implicated in the serotonin production pathway [101].

Hence, it is possible that serotonin production in vivo is diminished by an ALA deficit. In light of the above-presented data, supplementation with ω-3 PUFA and ALA might well have antidepressant-like effects owing to their anti-inflammatory qualities and involvement in multiple metabolic pathways. A summary of the antioxidant and hypolipemiant properties of *Perilla frutescens,* as derived from studies on human subjects, is presented in Table 3.

## 7. Miscellaneous Effects

In vitro and ex vivo studies clearly showed that the proprietary *Perilla frutescens* extract combines prokinetic and antispasmodic, as well as anti-inflammatory effects [102,103]. These actions make *Perilla frutescens* a perfect alternative therapeutic agent for gastrointestinal discomfort, as demonstrated in a double-blind, randomized, placebo-controlled parallel study by Buchwald-Werner et al., which enrolled 50 adult patients with gastrointestinal symptoms, such as cramps and constipation. The study found that *Perilla frutescens* extract significantly improved all gastrointestinal symptoms over time during the intervention stage [104]. A recent literature review by Thumann et al. outlined that human gut bacteria are able to metabolize rosmarinic acid and that it may have impacts on the gut microbiome, possibly acting as a prebiotic [105].

Lately, the need for active compounds derived from various herbs as potent antibacterial agents against a diverse spectrum of microorganisms has increased in order to combat human infection and preserve food [106,107]. The antibacterial efficacy of a polyphenol-rich *Perilla frutescens* extract against oral *Streptococci* and *Porphyromonas gingivalis* was investigated. The ethyl acetate extracts have significant antibacterial action against oral *Streptococci* as well as against *Porphyromonas gingivalis* variant [108]. The ethanolic extract of defatted *Perilla* seeds, on the other hand, inhibited the growth of oral pathogenic bacterial strains rather moderately, and in the study of Yamamoto et al. [108], among the polyphenols evaluated, luteolin showed significant antibacterial efficacy against oral bacteria.

The anti-fungal effects of *Perilla frutescens* oil against *Trichophyton mentagrophytes* were described [109], and it was shown that it produces a dose-dependent inhibition of various toxins in both methicillin-sensitive and -resistant *Staphylococcus aureus* [110]. The same *Perilla frutescens* type of extract was also investigated in terms of anti-fungal properties directed against a wide variety of fungi, and it manifested potent activity against *Aspergillus* spp. [41].

Just a few experiments have investigated *Perilla frutescens’* toxic capabilities. In Japan, occupational contact dermatitis is a well-known ailment among persons who come into contact with this plant [111]. Furthermore, in Korea, anaphylaxis caused by *Perilla frutescens* seeds [112] and occupational asthma produced by inhaling smoke from roasting these seeds [113] with an immunoglobulin E-mediated mechanism were documented.

## 8. Conclusions

This review outlined the proven therapeutical effects of *Perilla frutescens* extracts using data provided by trials performed on human subjects. Although the previous literature comprises three literature reviews on the phytochemical and phytopharmacological properties of *Perilla frutescens*, this paper is the first one that evaluated the clinical evidence for its effects.

The main perspectives that can be contoured from the presented literature evaluation are an important clinical effect of *Perilla frutescens* extracts on allergic rhinoconjuctivitis, especially in young populations, a potent hypolipemiant effect that, in conjunction with increased serum biological antioxidant potential, determines significant improvements in cognitive function, and a wide variety of miscellaneous clinical effects that need further exploration.

The inclusion of nutraceutical elements in personalized diets or as supplements, with a consistent regime of administration, represents another promising research perspective. The current literature review outlined the need for evaluating specific types of *Perilla frutescens* extracts, particularly in clinical scenarios where inflammation and/or allergic responses are present, in order to provide supplementary scientific data for supporting their therapeutic effects.

Rosmarinic acid is the most studied compound from *Perilla frutescens* extracts and constitutes support for the findings in the majority of clinical data. Although evidence from animal studies and cell cultures stipulates a wide variety of chemical compounds responsible for different therapeutic effects, data from clinical trials result only from a few substances comprised in various plant associations, and supplementary research is needed in order to demonstrate their therapeutic effects in controlled clinical settings.

Currently, there is not enough clinical evidence to be included in a systematic review and meta-analysis. This affirmation is supported by the fact that the scientific literature comprises only studies with small cohorts of human subjects, data in those studies are heterogeneously reported, so that a standardized approach would be difficult, incomplete reports or studies with a great degree of bias concerning patients’ selection, use of index tests and reference standards, as well as restrictive timeframes for proper evaluation of drug effects.

Given the plethora of in vitro and animal studies that emphasized the multiple biological effects of *Perilla frutescens*, it would be useful to assess its true limitations and possibilities in human subjects affected by specific disorders in a controlled experimental environment.

*Perilla frutescens* remains a focus point for researchers worldwide due to its complex phytochemical constituents, medicinal effects, and nutraceutical properties. Therefore, this review can serve as groundwork for subsequent studies on this plant.

## Figures and Tables

**Table 1:** Summary of evidence from in vitro and animal studies regarding the *Perilla frutescens* effects.

Activity	Part Used	Compound	Dose	Subject	Model	Reference
Anti-inflammatory and antiallergic effects	Leaves	AE + RA	AE—500 mg/kg RA—19 mg/kg	ddY mice	PCA response elicited by OVA	[27]
	Herb	Water decoction	500 mg/kg	Balb/c mice	PCA response elicited by OVA	[23]
	Seeds	AE	100 mL	C57BL/6 mice	Asthma provoked by OVA	[28]
	Seeds	RA	0.7 or 1.4 mg	Endothelial cell and mice	HMGB1 expression and HMGB1-mediated regulation of immune activation	[29]
	Leaves	AE and Luteolin	1 mg for both	ICR mice	Induced ear edema	[30]
	Leaves	Luteolin	1, 5 and 10 μM	BV-2 microglial cells	Nitric oxide production is enhanced by lipopolysaccharides	[31]
	Leaves	RA	30% ethanol extract	Mice; human mast cells	Respiratory allergic manifestations	[32]
	Leaves	AE	0.01 g/kg	SD rats, Rat peritoneal mast cell	Allergic response caused by anti-DNP IgE	[21]
	Leaves	Glycoprotein from the hot water extract	0.5 mg/mL	Rat peritoneal mast cell	Induced histamine release	[22]
	Leaves	Methanol extract	5–50 μg/mL	Human bronchial epithelial cells	Induced allergen gene expression	[33]
	Leaves	RA	Cell culture—0–2 μM Mice—1.4 mg/12 h	HUVECs, C57BL/6 mice	Production and interaction of inflammatory cytokines	[34]
	Leaves	RA	1.5 mg/24 h	C3H/He mice	Induced allergic asthma	[25]
	Leaves	Nine triterpene acids from ethanol extract	Inhibitory dose—0.09–0.3 mg	ICR mice	Ear inflammation	[35]
	Leaves	AE	400 μL	ICR mice	Ear edema	[36]
	Leaves	AE	1 mg b.i.w.	C3H/He mice	Influence over TNF-a production	[37]
Antioxidant and hypolypemiant effects	Leaves	AE	1.7 or 4.6 mg/mL	HUVECs, healthy female volunteers	Induced lipid oxidation	[38]
	Seeds	RA	20 mg/mL	H9c2 cardiac muscle cells	Induced programmed cell death	[15]
	Seeds	RA	10–50 μM	RAW 264.7 cells	Induced lipid production	[26]

Table legend: AE—aqueous extract; RA—rosmarinic acid; OVA—ovalbumin; PCA—passive cutaneous anaphylaxis; LPS—Lipopolysaccharides; TNF—tumor necrosis factor; IL—interleukin; EPCR—endothelial protein C receptor; LDL—low-density lipoprotein; NO—nitric oxide.

**Table 2:** Summary of evidence from studies performed in human subjects regarding the *Perilla frutescens* anti-inflammatory and antiallergic effects.

Disease	Number of Patients	Compound	Dose	Time-Frame	Results	Reference
Allergic rhinoconjunctivitis (AR)	128 children (Lertal Group-LG: 64 patients; Observation Group-OG: 64 patients)	Lertal: Quercetin 150 mg, *Perilla frutescens* 80 mg (as dry extract of the seeds containing rosmarinic acid, luteolin, apigenin and chrysoeriol), and Vitamin D3 5 mcg (200 IU).	1 tab/day	4–12 weeks	- Reduced the risk of AR exacerbation (LG) - Reduced number of days with the use at least one rescue medication (LG) - Higher cumulative days treated with rescue medication (OG)	[69]
Allergic rhinoconjunctivitis (AR)	146 children (LG+ standard treatment: 73 patients; OG+ standard treatment: 73 patients)	Lertal	1 tab q.d.	Baseline, after 2 and 4 weeks	- reduced TSS after 4 weeks (LG + OG) - 24 patients had symptom worsening between W2 and W4: 8 (LG)/16 (OG)	[67]
Seasonal allergic rhinoconjunctivitis (SAR)	23 adults (16 women, and 7 men), without control group	Lertal	1 tab b.i.d.	Baseline and after 1 month	- reduction of the overall symptoms - reduction in use of anti-allergic drugs	[70]
Seasonal allergic rhinoconjunctivitis (SAR)	Rosmarinic acid 50 mg (9 patients) Rosmarinic acid 200 mg (10 patients) Placebo (n10 patients)	Rosmarinic acid	50 or 100 mg q.d.	21 days	- Reduction of the specific and total symptoms. - Reduction of the numbers of inflammatory cells in nasal lavage fluid.	[24]
Atopic dermatitis (AD)	21 patients (14 women and 7 men)	Rosmarinic acid cream	0.3%, topical application b.i.d	8 weeks	- reduction of erythema on antecubital fossa (4 and 8 weeks) - reduction in the transepidermal water loss of the antecubital fossa (8 weeks). - Improvement of the specific and total AD symptoms.	[71]

**Table 3:** Summary of evidence from studies performed in human subjects regarding the *Perilla frutescens* antioxidant and hypolipemiant effects.

Main Outcome(s)	Number of Patients	Compound	Dose	Time-Frame	Results	Reference
LDL oxidation and antioxidant enzyme expression	8 healthy female volunteers	Red *Perilla frutescens* extract	120 mL single dose	Plasma at baseline, 30 min, 1, 2, and 4 h	- longer LDL oxidation lag times - lowered lipid peroxide formation, and the electrophoretic mobility of LDL	[38]
Mental function, fatty acid profile, biological antioxidant potential	32 healthy elderly volunteers (17 women, and 15 men)	*Perilla frutescens* seeds oil (PO) *Anredera cordifolia* (AC) leaf powder	PO: 1.47 mL q.d. PO + AC: 1.47 mL of PO and 1.12 g of AC	Baseline, and at 12 months	- higher cognitive index scores (POAC) - increased ALA and eicosapentaenoic acid levels, serum biological antioxidant potential (POAC) - hypolipemiant effect	[87]
Cognitive function	49 healthy elderly (24 men and 25 women)	*Perilla frutescens* seeds oil (PO) Nobiletin-rich air-dried immature ponkan powder (PP)	PO: 1.47 mL (0.88 g of ALA) q.d. PO + PP: 1.47 mL of PO and 1.12 g ponkan powder (2.91 mg of nobiletin)	Baseline, and at 12 months	- higher cognitive index scores (POPP) - increases in ALA, docosahexaenoic acid, BDNF levels - increase pf biological antioxidant potential	[88]
Biological Antioxidant Potential	PO group (n = 42 patients) Control group (n = 33 patients)	*Perilla frutescens* seeds oil (PO)	PO: 7.0 mL of PO q.d. Control: 7.0 mL of canola oil q.d.	Baseline, and at 12 months	- increased biological antioxidant potential and α-linolenic acid levels (PO) - improved cognitive function (PO group).	[89]
Mental condition	PO group (n = 38 patients) Placebo group (n = 37 patients)	*Perilla frutescens* seeds oil (PO)	PO: 7.0 mL of PO q.d. Control: 7.0 mL of olive oil q.d.	Baseline, and at 12 months	- reduced depression and apathy scores (PO) - decreased norepinephrine and serotonin levels (PO).	[71]

## Data Availability

The data presented in this study are available on request from the corresponding author. The data are not publicly available due to local policies.
